# CD24-Fc suppression of immune related adverse events in a therapeutic cancer vaccine model of murine neuroblastoma

**DOI:** 10.3389/fimmu.2023.1176370

**Published:** 2023-06-06

**Authors:** Xiaofang Wu, Priya Srinivasan, Mousumi Basu, Talia Zimmerman, Samuel Li, Yin Wang, Pan Zheng, Yang Liu, Anthony David Sandler

**Affiliations:** ^1^ The Joseph E. Robert Jr. Center for Surgical Care and The Sheikh Zayed Institute for Pediatric Surgical Innovation, Children’s National Hospital, George Washington University, Washington, DC, United States; ^2^ University of Maryland Medical Center, University of Maryland, Baltimore, MD, United States; ^3^ OncoC4. Inc, Rockville, MD, United States

**Keywords:** CD24Fc, tumor cell vaccine, neuroblastoma, immune-related adverse events, autoimmune profiling

## Abstract

**Introduction:**

The combination of Myc-suppressed whole tumor cells with checkpoint inhibitors targeting CTLA-4 and PD-L1 generates a potent therapeutic cancer vaccine in a mouse neuroblastoma model. As immunotherapies translate from pre-clinical to clinical trials, the potential immune-related adverse events (irAEs) associated with induction of potent immunity must be addressed. The CD24-Siglec 10/G interaction is an innate checkpoint that abrogates inflammatory responses to molecules released by damaged cells, but its role in cancer immunology is not well defined. We investigate irAEs of an effective whole cell neuroblastoma vaccine and subsequently the effect of CD24-Fc, a CD24 and Fc fusion protein, on both the vaccine efficacy and induced irAEs in a mouse neuroblastoma model.

**Methods:**

To test whether the whole tumor cell vaccination leads to autoimmune responses in other organ systems we harvested lung, heart, kidney and colon from naïve mice (n=3), unvaccinated tumor only mice (n=3), and vaccinated mice with CD24 Fc (n=12) or human IgG-Fc control (n=12) after tumor inoculation and vaccination therapy at day 30. The Immune cell infiltrates and immunogenic pathway signatures in different organ systems were investigated using NanoString Autoimmune Profiling arrays. Nanostring RNA transcript results were validated with immunohistochemistry staining.

**Results:**

The whole tumor cell vaccine combined with immune checkpoint therapy triggers occult organ specific immune cell infiltrates, primarily in cardiac tissue and to a lesser extent in the renal and lung tissue, but not in the colon. CD24-Fc administration with vaccination partially impedes anti-tumor immunity but delaying CD24-Fc administration after initial vaccination reverses this effect. CD24-Fc treatment also ameliorates the autoimmune response induced by effective tumor vaccination in the heart.

**Discussion:**

This study illustrates that the combination of Myc suppressed whole tumor cell vaccination with checkpoint inhibitors is an effective therapy, but occult immune infiltrates are induced in several organ systems in a mouse neuroblastoma model. The systemic administration of CD24-Fc suppresses autoimmune tissue responses, but appropriate timing of administration is critical for maintaining efficacy of the therapeutic vaccine.

## Introduction

Cancer immunotherapy in the form of checkpoint inhibitors targeting CTLA-4, PD-L1 and PD-1 has made significant impact in the treatment of solid tumors. However, immune-related adverse events associated with immunotherapy are and will be a significant problem as even more effective immunotherapeutic approaches are developed (1–3). Immune related adverse events (irAEs) are reported in multiple organ systems, often leading to profound pathology. Hypophysitis is observed in patients treated with ipilimumab (an anti-CTLA-4 inhibitor) (4), thyroid dysfunction is noted following pembrolizumab (a PD-1 receptor antibody) treatment (5) and gastrointestinal irAEs are common with any check-point inhibitor (CPI) (6). The specific presentation of the irAE and the severity of the event during treatment are unpredictable which in some cases can be fatal. The mechanism of irAE is not clearly understood and the ability to reduce these events without impeding anti-tumor immunity is and will be critical. Success in reducing irAE could have a transformative impact on the field of cancer immunotherapy.

Immunotherapy is thought to promote immunogenic tumor cell death that initiates antitumor immunity, but cell death may also induce damage-associated molecular patterns (DAMPs). These DAMP molecules recruit and activate dendritic cells (DCs) that present tumor-specific antigens to T cells for elimination of neoplastic cells, but may also lead to severe tissue damage (7). In order to limit non-specific tissue damage, the CD24-Siglec signaling pathway suppresses inflammation triggered by DAMPs through blockade of NF-κB activation (8). CD24 is a multifunctional molecule, with a wide distribution in diverse cell lineages including tumor cells and immune cells. It partners with its receptor, Siglec-10 in humans or Siglec-G in mice to abrogate inflammatory responses to molecules released by damaged cells (9–11). Recently a CD24-Fc fusion protein composed of the extracellular part of CD24 and human IgG1Fc has been administered to prevent acute graft-versus-host disease (GVHD) in a Phase 2a clinical trial in adult leukemic patients who underwent hematopoietic cell transplantation (12). In another clinical trial CD24-Fc successfully improved overall survival and reduced multiple organ autoimmune injury in treating severe COVID-19 patients (13). Thus, engaging CD24-signaling may provide protection from inflammation and/or autoimmune disease following acute tissue injury induced by effective cancer immunotherapy. In contrast to these potential beneficial effects, CD24 is overexpressed in ovarian cancer and breast cancer, and acts as a “don’t eat me” signal to protect cancer cells from phagocytosis that in turn promotes immune evasion (14). CD24 is thought to induce tumor progression by activating signaling molecules involved in proliferation and survival of cancer cells (15) and by increasing tumor suppressor activity of p53 (16). Blockade of CD24 and its receptor Siglec-10 reduces tumor growth and extends patient survival (14). Furthermore, CD24 on antigen presenting cells can act as a CD28 independent costimulatory molecule for activation of both CD4 and CD8-T cell responses (17–19). These conflicting observations in innate and acquired immunity highlight the crossover between tumor proliferation, antitumor immunity and autoimmunity suggesting that the predominant effects may be determined by the context in which CD24 is engaged as well as by glycosylation of CD24 (16).

Amplification of the Myc oncogene is associated with immune privilege in neuroblastoma (20). Targeting Myc *in vitro* with small molecule inhibitors induced Neuro2a tumor cell immunogenicity and enabled the production of a whole cell tumor vaccine in mouse tumor models (20). When the vaccine is combined with immune checkpoint inhibitors (anti-CTLA4 and anti-PD-L1 antibodies) potent tumor specific immunity is induced in the neuroblastoma mouse model (20). Although mice appear well and survive both the tumor challenge and the vaccine treatment, it is unknown if this tumor vaccine strategy induces autoimmune disorders in the host mice. In this study, we examined the autoimmune consequences of effective tumor vaccination on multiple organ systems and tested the impact of targeting the CD24-Siglec signaling pathway on both the vaccine therapy and autoimmune effects in the mouse neuroblastoma model. Nanostring autoimmune profiling of several organ systems indicated that the vaccine strategy induced moderate to severe lymphocytic infiltration and enhanced autoimmune signals in cardiac tissue and to a lesser extent in renal tissue. The lungs also demonstrated limited autoimmunity, but surprisingly the colon was essentially spared from the auto-immune effect of the vaccine therapy. Administration of CD24-Fc with the initial tumor vaccine dampened the therapeutic effect of the vaccine. When CD24-Fc administration lagged several days behind initial vaccination, vaccine efficacy was not altered and treatment resulted in excellent tumor free survival. Notably, CD24-Fc administration suppressed the autoimmune response detected in cardiac and renal tissues. These findings suggest that an effective whole cell vaccine in a mouse neuroblastoma model induces organ specific autoimmune responses and when CD24- Fc is appropriately administered, the irAE can be reduced without exacerbating tumor growth or impeding tumor immunity.

## Materials and methods

### Animals

Female C57BL/6 and A/J mice aged 6 weeks were purchased from Jackson Laboratories (Bar Harbor, Maine, USA). All procedures were approved by the Institutional Animal Care and Use Committee (IACUC) of Children’s National Hospital, Washington, DC.

### Cells

The mouse neuroblastoma Neuro2a cell line (Sigma, St. Louis, Missouri, USA) was cultured using DMEM supplemented with 10% heat-inactivated fetal bovine serum (FBS, Sigma) and 100 IU/mL penicillin, 100 µg/mL streptomycin. All media and supplements were purchased from Thermo Fisher Scientific (Waltham, Massachusetts, USA).

### Antibodies and reagents

Anti (α)-mouse CTLA-4, α-mouse programmed death-ligand 1 (PD-L1), and mouse IgG2b isotype antibodies were purchased from BioXCell (West Lebanon, New Hampshire, USA). I-BET726 was purchased from Millipore Sigma (Burlington, Massachusetts, USA) and JQ1 was purchased from Tocris (Minneapolis, Minnesota, USA). TLR7/8 ligand (Resiquimod, R848) was purchased from InvivoGen (San Diego, California). CD24-Fc and human IgG1-Fc were supplied by OncoImmune, Inc.

### Mouse tumor therapeutic models

As described previously (20), the right flanks of A/J mice were injected (subcutaneously) with 2×10^6^ Neuro2a cells on day 0. For vaccination, 2×10^6^ Myc inhibitor (BET/JQ1) treated and irradiated (40 Gy) Neuro2a cells were injected (subcutaneously) into the left flank of each mouse on day 7, 10 and 13 as a whole tumor cell vaccine along with anti-CTLA-4 (aCTLA4) and anti-PD-L1 (aPD-L1) antibodies (100 µg/mouse) administered intraperitoneally. TLR7/8 agonist (25 µg/mouse) was used on day 7 in an attempt to enhance immunity induced by the vaccine. CD24-Fc (100 µg/mouse) or IgG-Fc (100 µg/mouse) was administered intraperitoneally on day 7, 10 and 13 (immediate model) or on day 10, 13 and 16 (delayed model). Mice were monitored daily following tumor inoculation and tumor growth was recorded in two dimensions. Tumor volume was calculated using the following formula: largest diameter^2^ × smaller diameter × 0.52. A tumor size of 20 mm in any dimension was designated as the endpoint, and mice were euthanized at that time. All the procedures are approved by the IACUC at Children’s National Hospital and are in accordance with the humane care of research animals.

### Nanostring

Lung, heart, colon and kidney were harvested from mice in four groups: 1.) naïve, 2.) unvaccinated tumor only, 3.) vaccinated with CD24-Fc or 4.) vaccinated with IgG-Fc on day 30 after tumor cell inoculation. RNA was extracted and gene expression was directly measured via counts of corresponding mRNA in each sample using an nCounter murine AutoImmune Profiling Panel (NanoString, Seattle, WA, USA). For full details, see our previous publication (20). Briefly, 100 ng of high-quality total RNA was hybridized with reporter probes, and then biotinylated capture probes at 65°C for 16–18 hr before being placed into the nCounter Prep station in which samples were affixed to a cartridge. Cartridges were then read by the nCounter Digital Analyzer optical scanner. Further advanced immune-profiling analysis was performed using nSolver 4.0 analysis software with nCounter advanced analysis package (NanoString Technologies). Genes were grouped into 14 immune cell types and 35 immune functions according to the manufacturer’s designation (20).

### Characterization of mouse cardiac tissue by immunohistochemistry

Mouse hearts were fixed in 10% neutral buffered formalin (pH 6.8–7.2; Richard-Allan Scientific, Kalamazoo, Michigan, US) for paraffin embedding and sectioning. Five μm tissue sections were cut with a microtome, and sample processing and IHC staining were performed as previously described (20) using rabbit polyclonal to CD45 antibodies (1:200. Abcam, Cambridge, Massachusetts, US). Isotype-matched antibodies were used for negative controls. Optical density (mean gray value) was obtained by color deconvolution analysis with Image J.

### Statistical analysis

Statistical analysis of nanostring gene expression, normalization, clustering, Pathview plots and fold-changes were performed using the Advanced Analysis Module in the nSolver™ Analysis Software version 4.0 from NanoString Technologies (NanoString Technologies, WA, USA) following our published method (20). Briefly, raw data for each sample were normalized to the geometric mean of housekeeping genes using the geNorm algorithm. Pathway scores were calculated as the first principal component (PC) scores for each sample based on the individual gene expression levels for all the measured genes within a specific pathway. The cell type score is calculated as the mean of the log2 expression levels for all the probes included in the final calculation for that specific cell type. An increase of 1 corresponds to a doubling in abundance (nanostring.com). All differentially expressed genes were subjected to KEGG term analysis, with significance accepted at p < 0.05. The Benjamini-Yekutieli method was used to control the false discovery rate. All statistical analyses of nanostring data were carried out in R v3.4.3 software.

Statistical significance for each set of experiments was determined by the unpaired 2-tailed Student’s t-test, and the specific tests were indicated in the figure legends. The data are expressed as the mean ( ± SD), with p<0.05 considered statistically significant. For the survival analysis, we calculated and compared the median survival time and the cumulative survival probability using the Kaplan-Meier survival estimator followed by a log-rank test, and calculated hazard ratio (HR) using the Cox proportional-hazards regression model.

## Results

### Inoculation of a whole tumor cell vaccine combined with immune checkpoint therapy triggers autoimmune responses and immune cell infiltrates primarily in cardiac tissue and with limited infiltrates in renal and lung tissue

**Figure 1:**
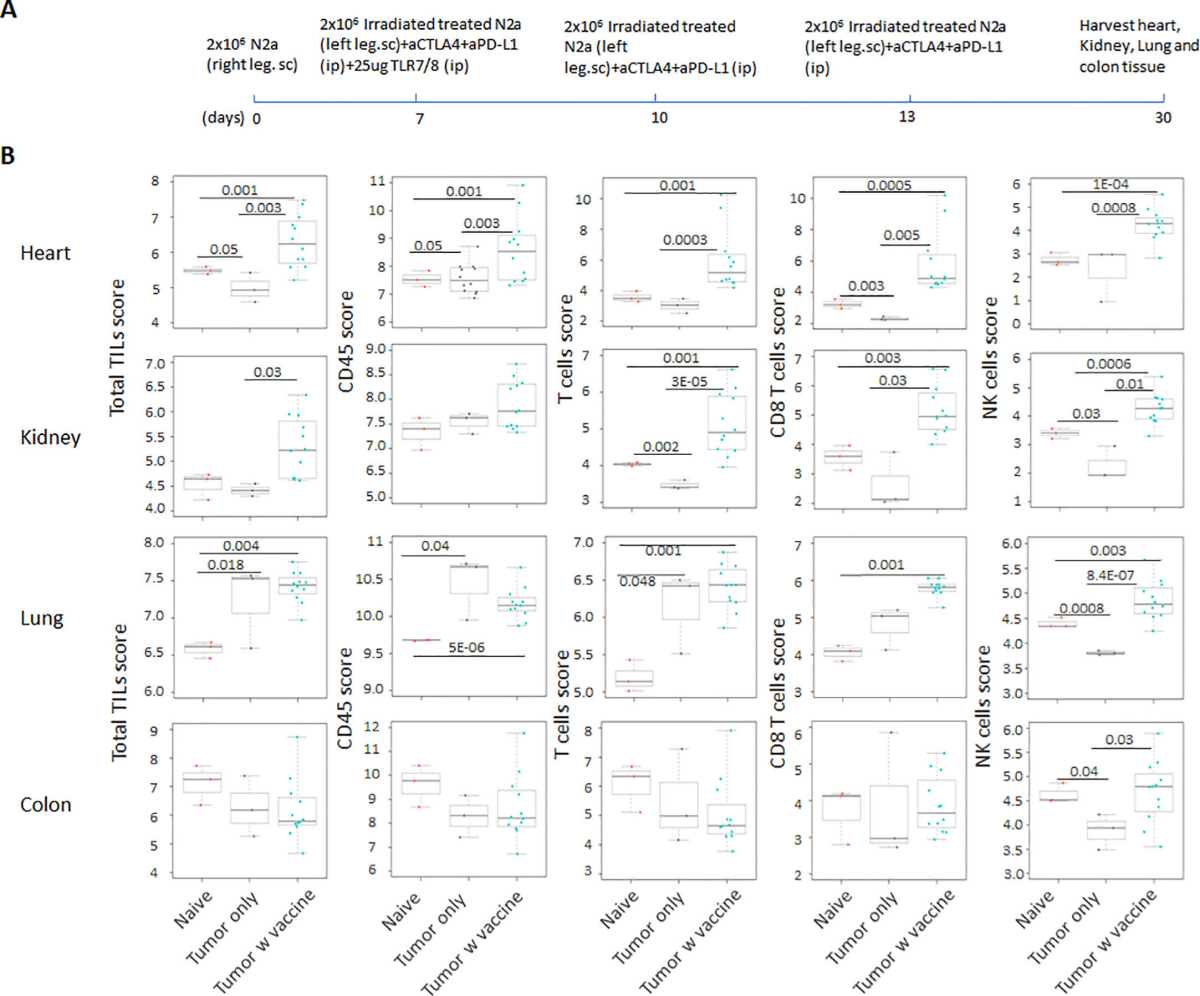
**(A)** Schematic design of tumor cell vaccination. To test whether whole tumor cell vaccination leads to autoimmune responses in other organ systems, we injected (subcutaneously) the right flanks of A/J mice with 2×10^6^ Neuro2a cells on day 0. For vaccination, 2×10^6^  Myc inhibitor (BET/JQ1) treated and irradiated (40Gy) Neuro2a cells were injected (subcutaneously) into the left flank of each mouse on day 7, 10 and 13 as a whole tumor cell vaccine along with anti-CTLA-4 and anti-PD-L1 antibodies (100 µg/mouse) administered intraperitoneally. TLR7/8 agonist (25 µg/mouse) was used on day 7. **(B)** The lung, heart, kidney and colon were harvested from naïve mice (n=3), unvaccinated tumor baring mice (n=3), and vaccinated mice (n=12) after tumor inoculation at day 30. The global expression of mRNA from each organ was investigated using NanoString Autoimmune Profiling arrays. Nanostring Autoimmune Profiling analysis revealed that heart, kidney and lung from the vaccination group demonstrated a moderate to severe increase in signature markers for total TIL, CD45 cells, T cells, CD8+ cells and NK cells when compared to naïve and tumor only control mice. Box plots show distribution of immune cells by relative number present within mouse heart, kidney, lung and colon calculated by gene expression. (p<0.05). As abundance estimates (cell type scores) are calculated in log2 scale, an increase of 1 on the vertical axis corresponds to a doubling in abundance. The horizontal black line on the box plot represents the median expression, and each symbol represents a single individual. Statistical significance was determined by unpaired two-tailed Student’s t-test, and p<0.05 was considered statistically significant.

**Figure 2:**
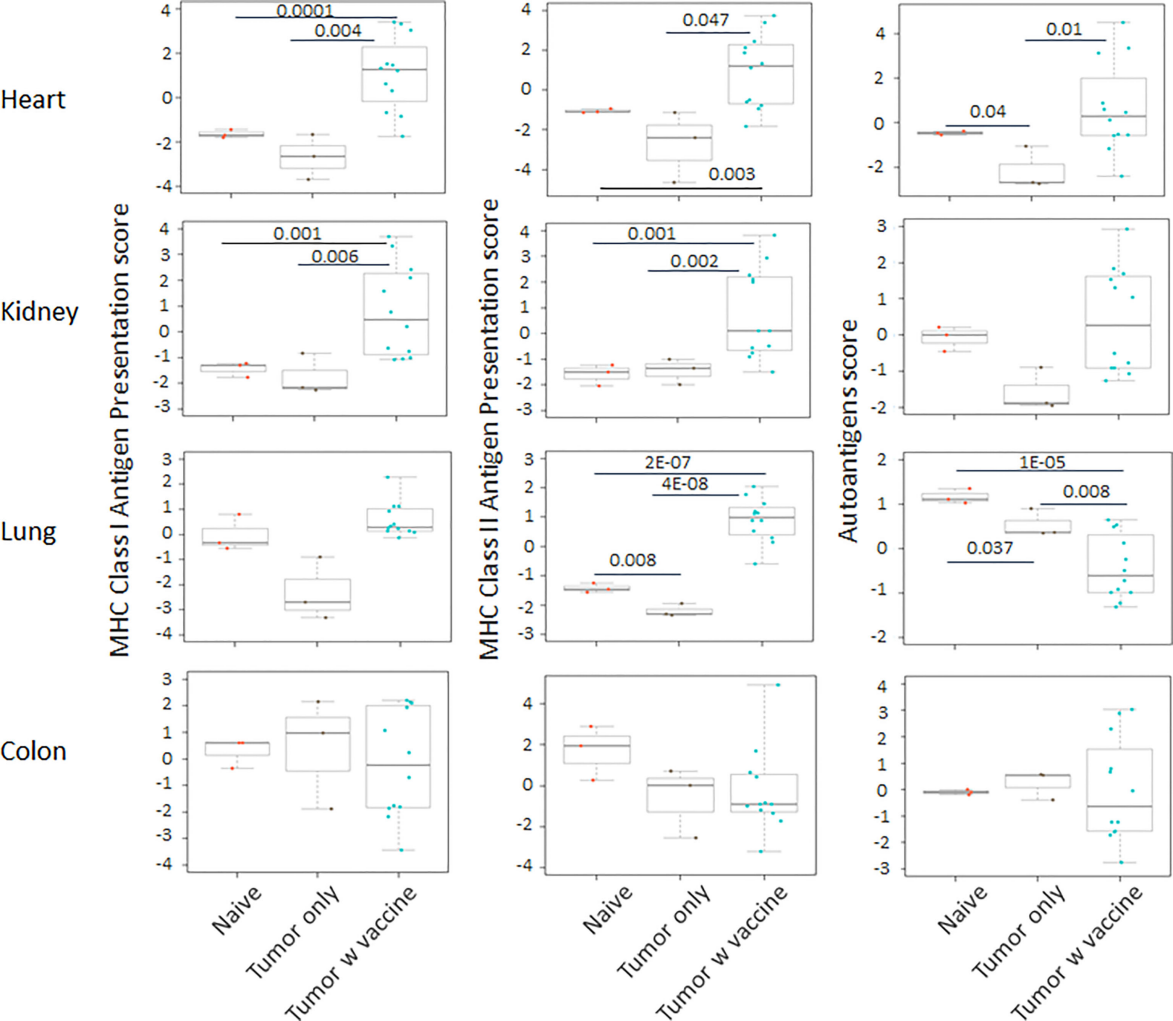
The pathway scores of multiple autoimmune signals that relate to antigen presentation were markedly enhanced in the tumor vaccinated group (n=12) in heart, kidney and lung, but not in the colon when compared with the organs that were collected from unvaccinated tumor only mice (n=3) and naïve mice (n=3) at day 30 after tumor inoculation. The vaccination strategy is described in **Figure 1**. Unpaired two-tailed Student’s t-test was performed for the statistical analysis, and p<0.05 was considered statistically significant.

**Figure 3:**
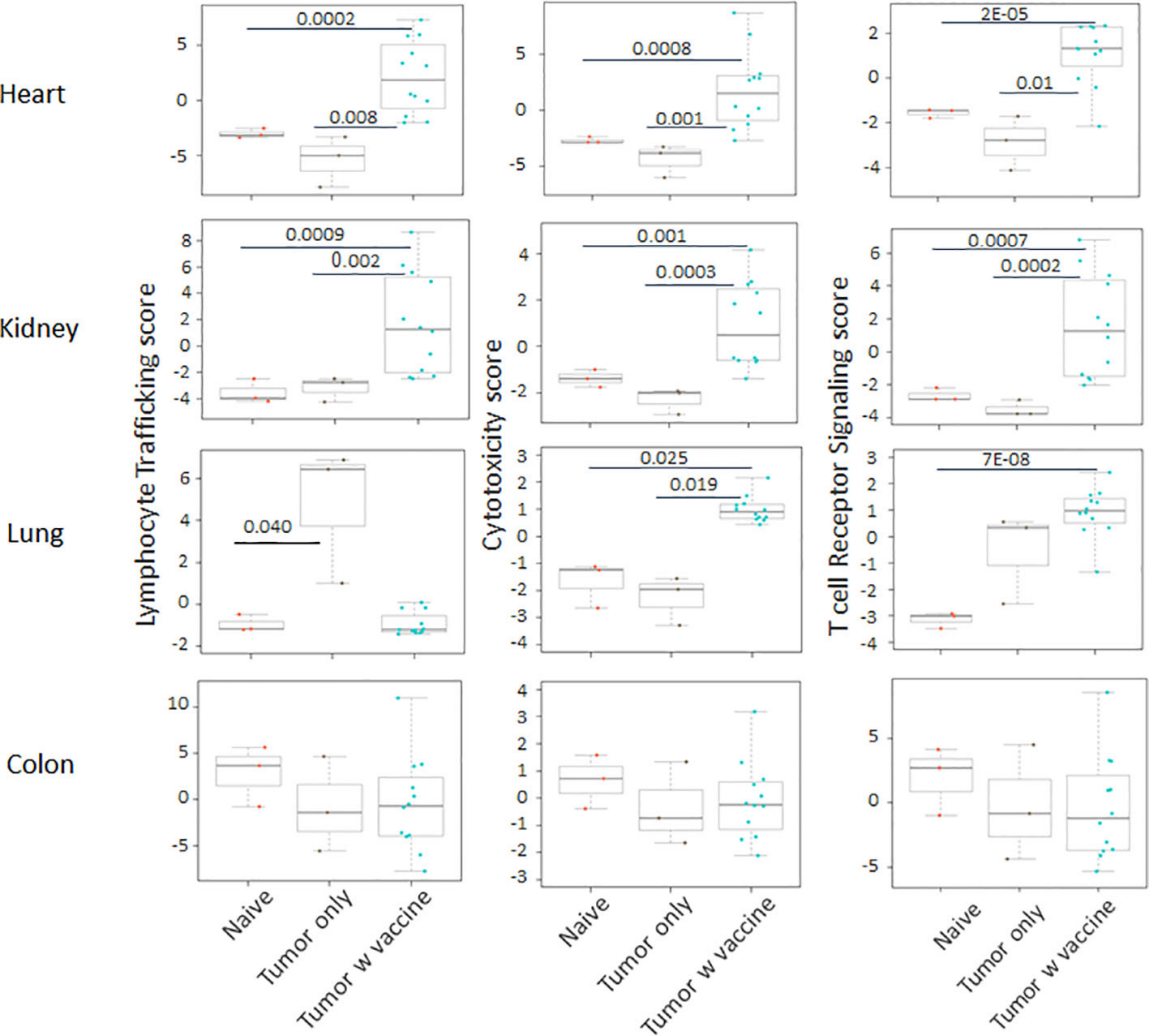
The autoimmune signaling pathway scores that relate to lymphocyte trafficking were all significantly increased in the tumor vaccinated group in heart, kidney and lung, but not in colon when compared with the organs collected from unvaccinated tumor only mice (n=3) and naïve mice (n=3) at day 30 after tumor inoculation. The vaccination strategy is described in **Figure 1**. Unpaired two-tailed Student’s t-test was performed for the statistical analysis and p<0.05 was considered statistically significant.

**Figure 4:**
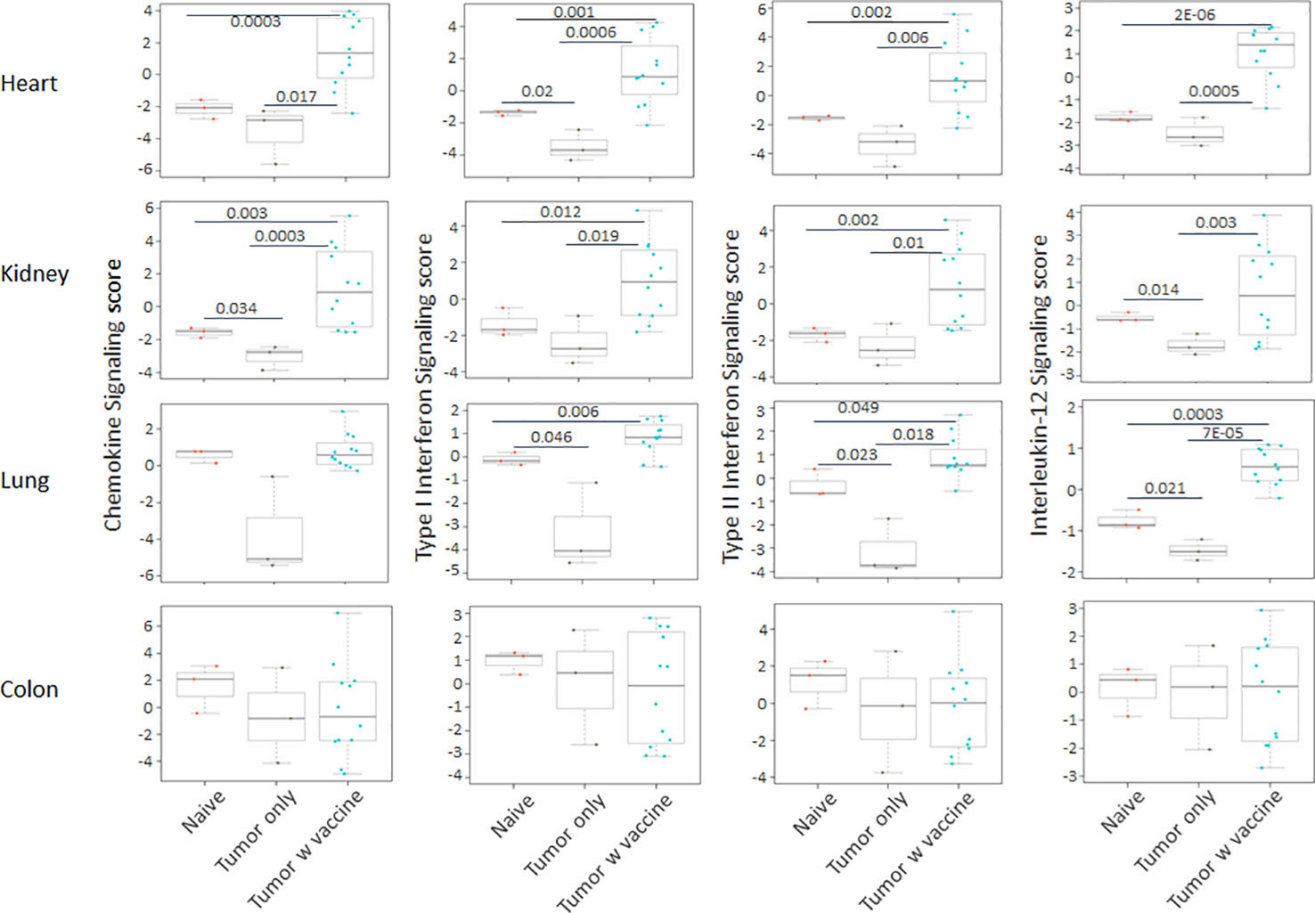
The autoimmune signaling pathway scores that related to chemokines and cytokines were also markedly augmented in the heart, kidney and lung tissue collected from the tumor vaccinated mouse group, but this change was absent in the colon when compared with the organs that were collected from unvaccinated tumor only mice (n=3) and naïve mice (n=3) at day 30 after tumor inoculation. The vaccination strategy is described in **Figure 1**. Unpaired two-tailed Student’s t-test was performed for the statistical analysis and p<0.05 was considered statistically significant.

Prior work from our laboratory shows that a neuroblastoma vaccine strategy containing BET/JQ1 treated cancer cells combined with anti-CTLA4 and anti-PDL-1 checkpoint inhibitors induced robust anti-tumor immunity and cured mice with established tumors (20). To test whether this whole tumor cell vaccination leads to autoimmune responses in other organ systems, we injected 2×10^6^ WT Neuro2a cells subcutaneously on day 0 on the right leg of A/J mice. For vaccination, 2×10^6^ Myc inhibitor (BET/JQ1) treated and irradiated (40 Gy) Neuro2a cells were injected (subcutaneously) into the left flank of each mouse on day 7, 10 and 13 as a whole tumor cell vaccine along with anti-CTLA-4 and anti-PD-L1 antibodies (100 µg/mouse) administered intraperitoneally. TLR7/8 agonist (25 µg/mouse) was used on day 7. The Schematic experimental design was shown in **Figure 1A**. We harvested lung, heart, kidney and colon from naïve mice (n=3), unvaccinated tumor only mice (n=3), and vaccinated mice after tumor inoculation and vaccination therapy at day 30 (n=12). The global expression of mRNA was investigated using NanoString Autoimmune Profiling arrays. Nanostring analysis revealed that the heart, kidney and lung from the vaccination group demonstrated a moderate to severe level of upregulation in the expression of signature markers for total TIL, CD45 cells, T cells, CD8+ cells, NK cells, dendritic cells, neutrophils, macrophages, and B cells when compared with naïve and tumor only control mice (**Figure 1B**; **Supplement Figure 1**). In addition, the scores of multiple autoimmune signaling pathways that related to antigen presentation (**Figure 2**), lymphocyte trafficking (**Figure 3**), chemokines and cytokines (**Figure 4**), and major inflammatory signaling pathways (**Supplemental Figure 2**), were all enhanced in the tumor vaccinated group. Interestingly, there was no significant autoimmune response detected following vaccination in the colons of the mice tested.

### CD24-Fc administration with vaccination can partially impede anti-tumor immunity, but delaying CD24-Fc therapy until after initial vaccination reverses this effect

**Figure 5:**
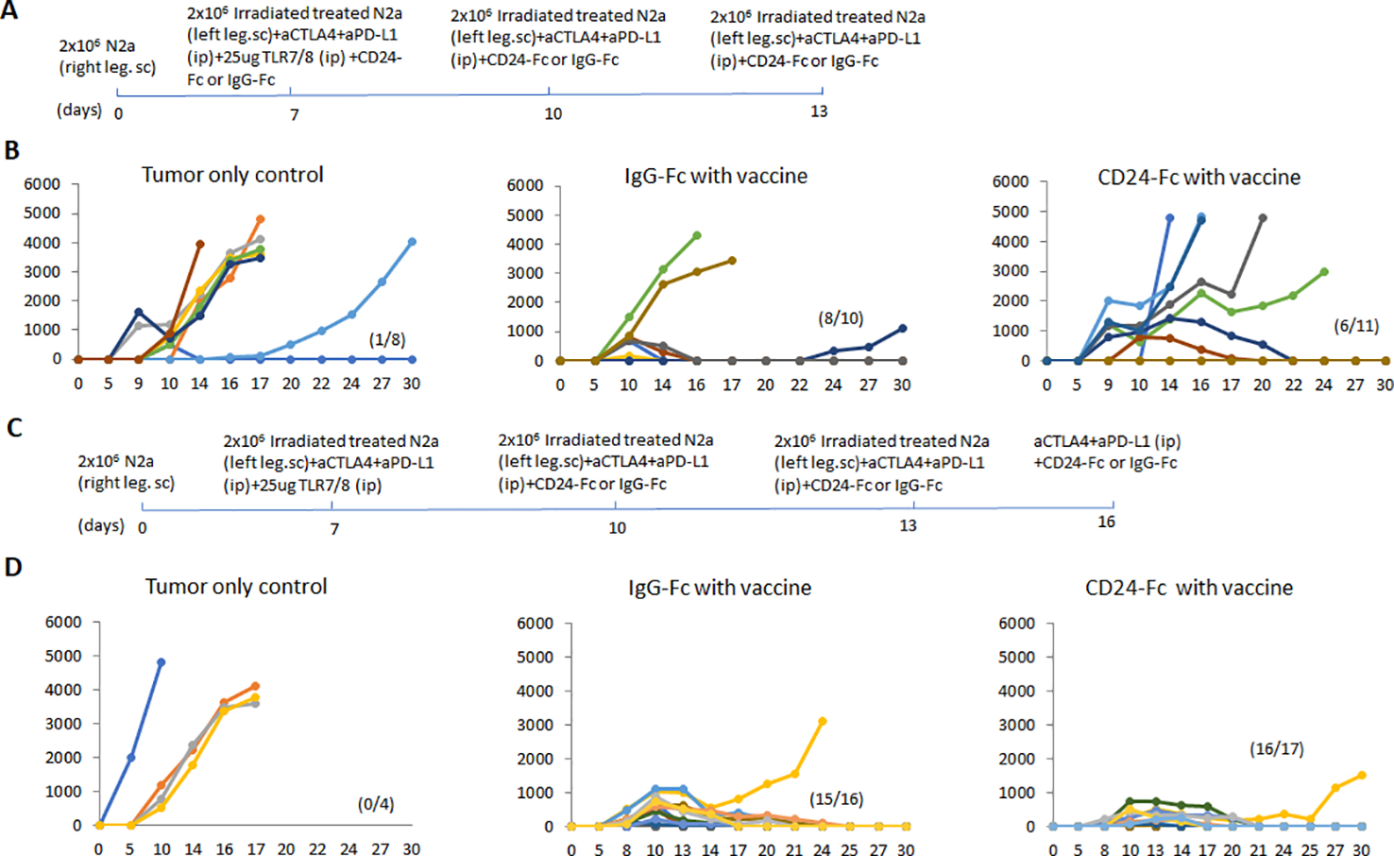
**(A)** The immediate vaccine model in which the vaccine protocol and timeline are depicted. 2×10^6^ Neuro2a cells were injected (subcutaneously) in the right flanks of A/J mice on day 0. CD24-Fc or human IgG-Fc control was injected simultaneously with the whole cell vaccine plus anti-PD-L1/CTLA4 antibodies on day 7, 10 and 13 post tumor inoculation. **(B)** The graphs reflect individual tumor growth in various treatment groups. Absence of tumor in individual mice is recorded in parenthesis. **(C)** The delayed vaccine model in which the vaccine protocol and timeline are depicted. The vaccination protocol and anti-PD-L1/CTLA4 antibodies were injected on day 7, 10 and 13, while CD24-Fc or IgG-Fc was administered three days after the first dose of vaccine on day 10, 13 and 16. **(D)** The graphs reflect individual tumor growth in the various treatment groups. Absence of tumor in individual mice is recorded in parenthesis.

**Figure 6:**
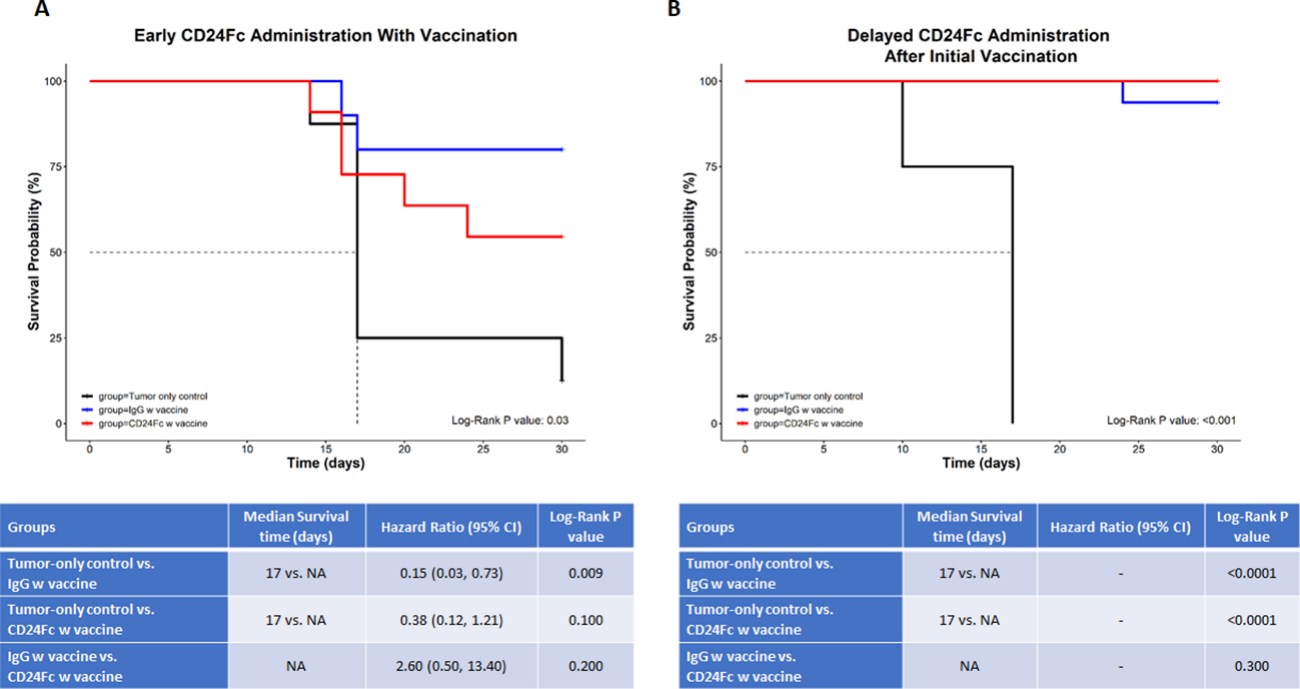
In this survival analysis, we compared survival and tumor growth between the three study groups (tumor-only control vs. IgG w vaccine vs. CD24-Fc w vaccine) **(A)** depicts the immediate (early) administration of CD24-Fc, while **(B)** depicts the delayed administration of CD24-Fc with vaccine. For the survival analysis, we calculated and compared the median survival time and the cumulative survival probability using the Kaplan-Meier survival estimator followed by a log-rank test, and calculated hazard ratio (HR) using the Cox proportional-hazards regression model. The comparison between the groups is shown in the tables below the graphs. * NA= median survival time could not be calculated since at least 50% of the subjects in that group didn’t have the outcome event (death) ** Hazard ratios could not be calculated due to a lack of adequate outcome events.

CD24 signaling is reported to promote immune evasion and tumor progression (14), yet this pathway is also thought to suppress autoimmunity through down regulation of DAMP signaling. In order to determine the effect on tumor immunity, we tested two neuroblastoma mouse models in which CD24-Fc was administered either at the time of tumor vaccination or three days following the initial vaccination to determine if the timing of CD24 signaling altered immunity. Briefly, 2×10^6^ WT Neuro2a cells were subcutaneously administered on day 0 on the right leg of AJ mice, then two treatment models were used. In the *immediate* vaccine model, CD24-Fc or human IgG-Fc control was injected simultaneously with the whole cell vaccine plus anti-PD-L1/CTLA4 antibodies at day 7, 10 and 13 post tumor inoculation (**Figure 5A**). In the second *delayed* vaccine model, vaccine and anti-PD-L1/CTLA4 antibodies were injected on day 7, 10 and 13, while CD24-Fc or IgG-Fc was administered three days after the first dose of vaccine, which was injected on day 10, 13 and 16 (**Figure 5C**). The methods and timelines are shown in **Figures 5A, C**. The individual tumor growth in various treatment groups from the early vaccine model and the delayed vaccine model were monitored and compared (**Figures 5B, D**). None of the mice developed tumor at the site of the vaccine cell injection. Significant therapeutic survival benefit was observed in vaccine groups either with CD24-Fc or with IgG-Fc when compared with tumor only control groups in both models, but the anti-tumor effect was more profound in the model of delayed CD24-Fc treatment (**Figure 6**). In the vaccine model, eight out of ten mice (80%) of the IgG/vac group were cured of the high dose 7 day established tumors, compared with tumor only control (p=0.009), whereas administration of CD24-Fc at the start of vaccination resulted in 55% cure, which was better than controls (13% cure) without vaccine, but was not statistically significant (p=0.1) (**Figure 6A**). In the second model of delayed CD24-Fc administration, there was no difference in tumor free survival between the mice that received IgG or CD24-Fc treatment (93% versus 94%), in which the tumor control group (no vaccine) had no survivors at day 30 (**Figure 6B**). In summary, these results suggest that CD24-Fc administration at the time of initial tumor vaccination can impair tumor immunity, but if the administration of CD24-Fc is delayed after the initial vaccination and administered with the boosters, the effect on tumor immunity induced by the vaccine does not appear to be hindered.

### CD24-Fc treatment with vaccination can ameliorate the autoimmune responses induced by effective tumor vaccination

**Figure 7:**
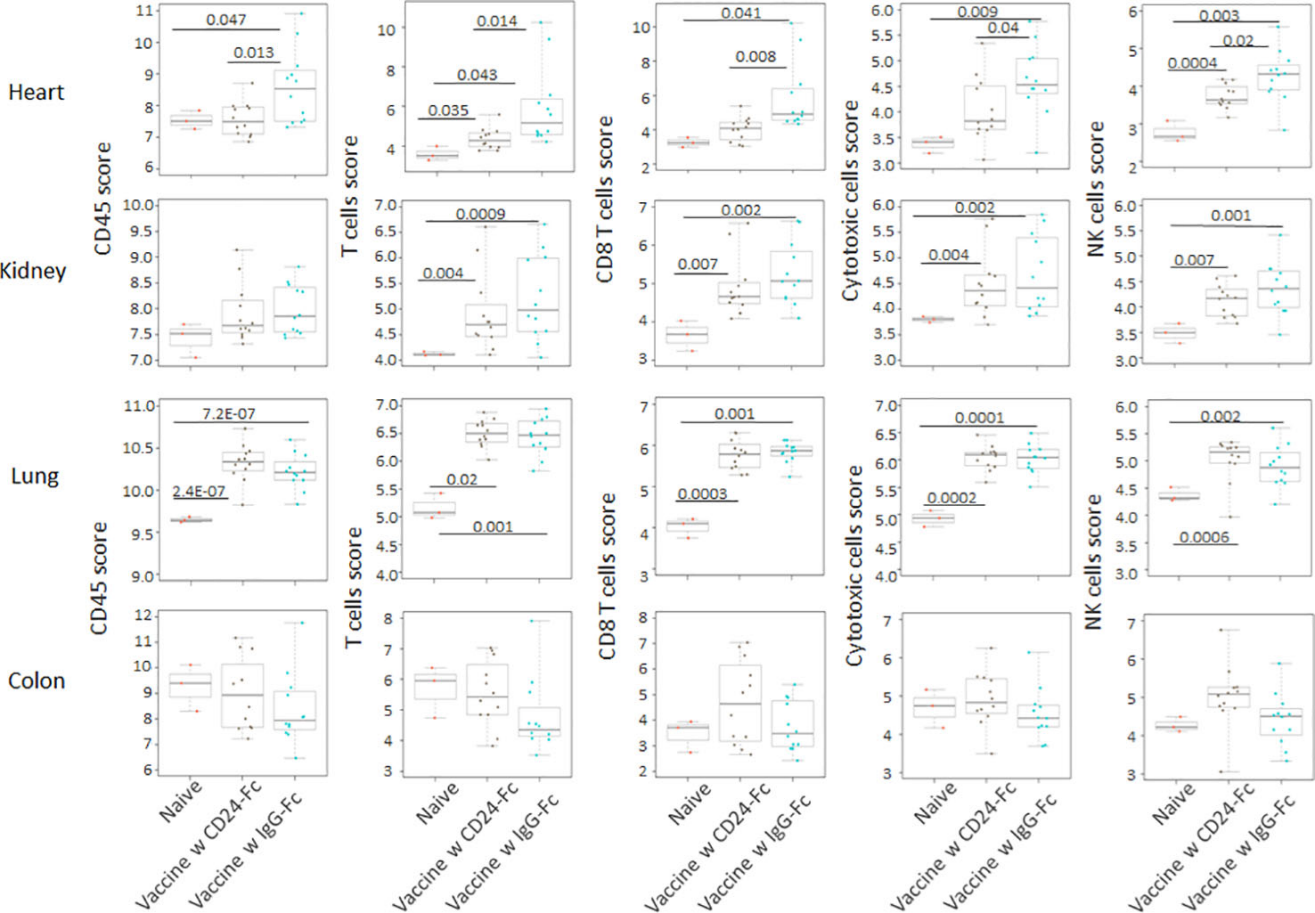
Following the delayed vaccination model in which the CD24-Fc or IgG-Fc was administered three days after the first dose of vaccine, the auto-immune responses in lung, heart, kidney and colon tissues of mice in the various groups, including naïve mice (n=3), vaccinated mice administered IgG-Fc with vaccination (n=12) and vaccinated mice administered CD24-Fc with vaccination (n=12) at day 30 after tumor inoculation were compared using NanoString Autoimmune Profiling arrays. Cell type analysis reveals that the influx of several immune cell subtypes was suppressed by delayed CD24-Fc treatment in cardiac tissue. Statistical significance was determined by unpaired two-tailed Student’s t-test and p<0.05 was considered statistically significant.

**Figure 8:**
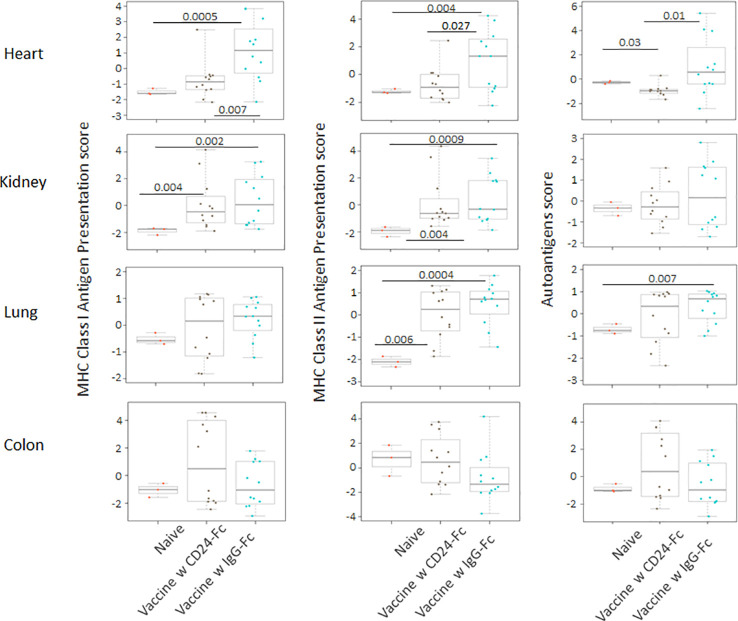
Delayed CD24-Fc treatment repressed the pathway scores of multiple autoimmune signaling that related to antigen presentation in cardiac tissue. Statistical significance was determined by unpaired two-tailed Student’s t-test and p<0.05 was considered statistically significant.

We analyzed auto-immune responses in lung, heart, kidney and colon tissues of mice in the various groups, including naïve mice (n=3), vaccinated mice administered IgG-Fc with vaccination (n=12) and vaccinated mice administered CD24-Fc with vaccination (n=12) at day 30 after tumor inoculation and vaccination therapy following delayed vaccine model. The global expression of mRNA was compared using NanoString Autoimmune Profiling arrays. The upregulated autoimmune signatures were broadly suppressed by CD24-Fc in heart tissue. Concurrent with changes in immune cell profiles (**Figure 7**; **Supplement Figure 3**), CD24-Fc repressed multiple genes that include antigen presentation (**Figure 8**), lymphocyte differentiation and trafficking (**Supplement Figure 4**), and chemokines and cytokines (**Supplement Figure 5**), major inflammatory signaling pathways (**Supplement Figure 6**) in the cardiac tissue assayed. Most genes that were significantly upregulated by tumor vaccine therapy were dampened by CF24-Fc. The top 28 up-regulated immune genes expressed in the heart tissue of vaccinated mice compared to naïve controls, were also amongst the most dampened genes suppressed by CD24-Fc (fold change>3 and p value<0.05). These genes are listed in **Table 1**. These results suggest that CD24-Fc can efficiently suppress the autoimmune response that was induced by vaccination therapy in cardiac tissue. There are also variable degrees of autoimmune response following vaccination in kidney and lung tissue samples, in which CD24-Fc suppressed specific targets, but without the broad impact it seemed to have in the cardiac tissues. (**Supplement Figures 4**–**6**). No significant autoimmune responses were detected in colon tissue following vaccination and CD24-Fc did not change autoimmune profiles in these tissue specimens either.

**Table 1:** Top statistically significantly up-regulated genes in heart tissue of vaccinated mice compared with naïve control, which are the most dampened genes by CD24 Fc (fold change>3 and p value<0.05,).

Gene ID	Fold change (Vaccinated vs Naïve)	P-value	Fold change (CD24Fc vs IgG w vaccination)	P-value	Gene Function
Cd8b1	71.4	9E-05	-11.7	6E-05	Lymphocyte Trafficking, T-cell Receptor Signaling
Cd6	31.7	7E-04	-17.3	2E-05	Lymphocyte Trafficking
Cd3d	26.9	2E-04	-12.5	1E-05	T-cell Checkpoint Signaling, T-cell Receptor Signaling
Cd27	24.0	6E-04	-16.7	7E-06	T-cell Checkpoint Signaling
Cd5	23.9	0.003	-19.4	3E-05	T-cell Checkpoint Signaling
Lat	21.9	0.003	-31.9	3E-06	Fc Receptors and Phagocytosis, Growth Factor Signaling, NF-kB Signaling, T-cell Receptor Signaling
Cxcl10	20.0	0.002	-4.1	0.009	Chemokine Signaling, Cytosolic DNA Sensing, Endothelial Activation, Th17 Mediated Biology, TNF Family Signaling, Toll Like Receptor Signaling
Cd4	19.5	0.003	-19.8	2E-05	Lymphocyte Trafficking, T-cell Checkpoint Signaling, T-cell Receptor Signaling
Zap70	19.2	0.003	-14.7	5E-05	NF-kB Signaling, T-cell Receptor Signaling
Cd3g	18.2	5E-04	-12.3	1E-05	T-cell Checkpoint Signaling, T-cell Receptor Signaling
Cd3e	17.1	5E-04	-11.4	1E-05	T-cell Checkpoint Signaling, T-cell Receptor Signaling
Itk	14.9	0.003	-7.6	5E-04	Chemokine Signaling, Fc Receptors and Phagocytosis, Lymphocyte Trafficking, T-cell Receptor Signaling
Xcl1	12.2	0.002	-4.9	3E-04	Chemokine Signaling
Ccl8	8.6	0.003	-3.5	0.005	Chemokine Signaling
Il2ra	8.2	0.019	-11.9	9E-05	Other Interleukin Signaling, Th2 Differentiation
Cd7	7.1	0.003	-3.1	0.002	Lymphocyte Trafficking
Lrr1	5.8	0.025	-9.6	7E-05	MHC Class I Antigen Presentation
Hist1h4k	5.1	0.008	-7.0	1E-05	Autoantigens
Slamf6	5.0	0.016	-3.0	0.007	Cytotoxicity
Gata3	4.5	0.02	-4.0	0.001	Th2 Differentiation
Hist1h3b	4.4	0.009	-5.6	2E-05	Autoantigens, Epigenetics and Transcriptional Regulation
Pycard	4.4	0.046	-3.2	0.013	Cytosolic DNA Sensing, Inflammasomes, NLR Signaling
Il18	4.0	0.044	-4.3	0.002	Cytosolic DNA Sensing, NLR Signaling, Other Interleukin Signaling
Kif22	3.9	0.036	-5.9	1E-04	MHC Class II Antigen Presentation
Ikzf1	3.8	0.026	-5.1	1E-04	Epigenetics and Transcriptional Regulation
Casp1	3.8	0.017	-3.3	0.001	Cytosolic DNA Sensing, Inflammasomes, NLR Signaling
Ikzf3	3.6	0.037	-5.1	2E-04	Epigenetics and Transcriptional Regulation
Hist1h2bk	3.6	0.013	-4.4	5E-05	Autoantigens

**Figure 9:**
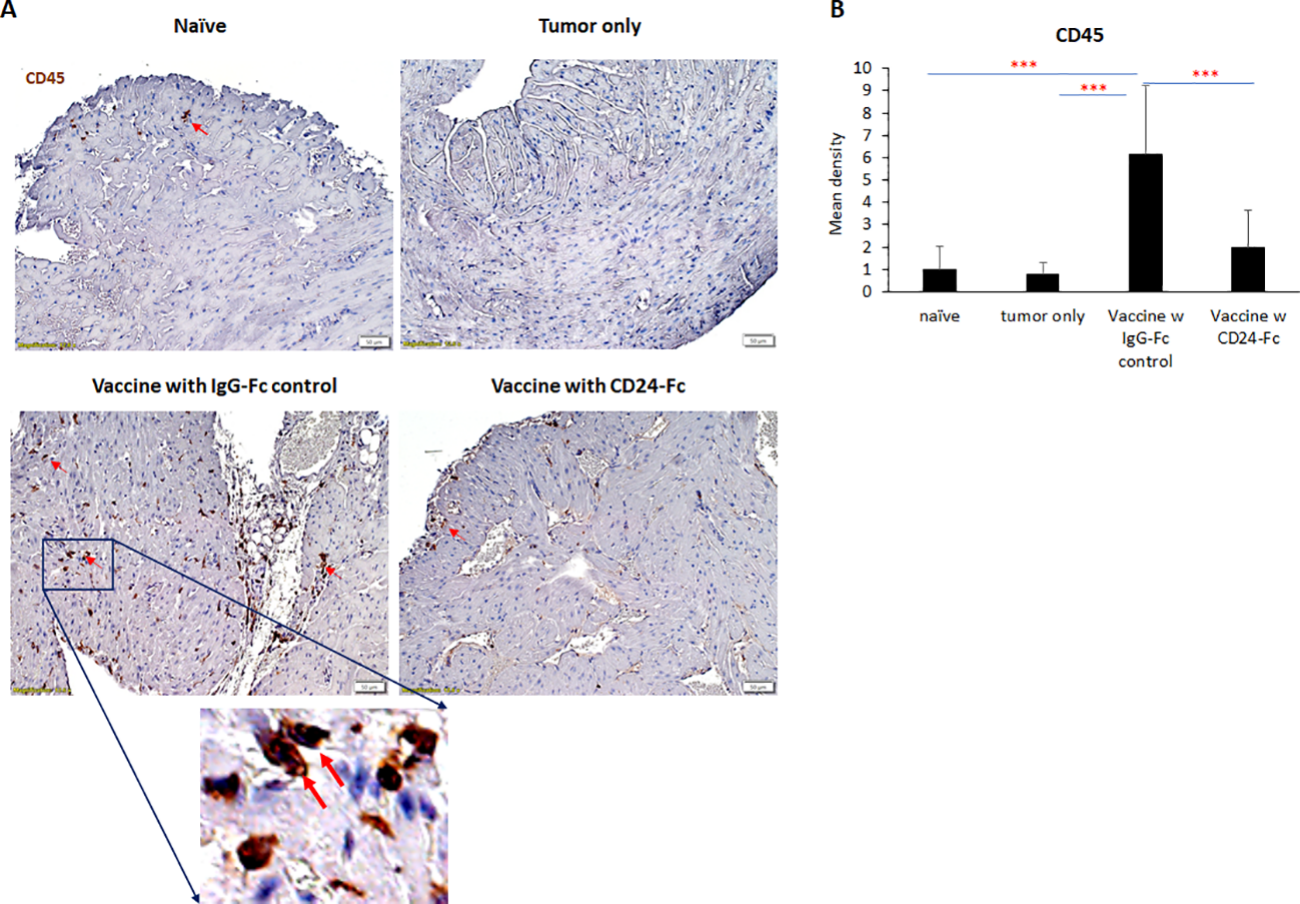
To validate nanostring results, CD45 expression was evaluated with immunohistochemical staining in heart tissue from naïve, tumor only control, and vaccination groups combined with either IgG-Fc or with CD24-Fc. **(A)** Representative images of CD45 staining visualized with diaminobenzidine **(DAB)** (brown) and hematoxylin (blue, nuclei) counter staining. Area in the blue box was enlarged and the CD45 staining of corresponding area was shown. **(B)** Optical density (mean gray value) obtained by color deconvolution analysis. Optical density graph bars represent the mean ± SD (n = 20 images). ***p<0.001, determined by unpaired two-tailed Student’s t-test.

To validate nanostring RNA transcript results, we performed immunohistochemistry staining of the immune cell marker CD45 on the same heart samples that were used for nanostring analysis. Results showed that the IgG-Fc group had significantly more CD45 (**Figure 9**) positive immune cell infiltrates than unvaccinated tumor only controls and naïve controls. Adding CD24-Fc significantly reduced the infiltration of CD45 positive cells in the cardiac tissue. Taken together, these data demonstrate that autoimmunity occurs to a different extent in various organ systems and the administration of CD24-Fc alleviates much of the autoimmune response observed. Despite dampening these immune related adverse tissue specific events, appropriate timing of CD24-Fc administration can still maintain sufficient immunotherapeutic effect of vaccination in the mouse neuroblastoma model.

## Discussion

The combination of Myc-inhibited tumor cells with checkpoint inhibition generates a potent therapeutic cancer vaccine in the mouse neuroblastoma model. We previously noted that a combination of both vaccination and checkpoint inhibitors cured 75% of mice and significantly improved long-term survival despite a large initial tumor cell challenge (20). To consider translating this therapeutic strategy from pre-clinical studies to clinical trials, knowledge of auto-immune side effects is essential. The great progress made by cancer immunotherapy is tempered by the occurrence of irAE. The immune related toxicities can be unpredictable, effect multiple organ systems and can be of variable severity. Managing these adverse events is a major challenge for the continued success of current immunotherapies or for the application of potent combined immunotherapeutic approaches. Several other aspects of irAE remain clinically concerning in that: i.) there may be an association between the efficacy of an anti-tumor immunotherapeutic response and the severity of the irAE (21, 22); ii.) many diagnostic challenges persist in patients with irAE in which some of the consequences of organ injury may be clinically occult; and iii.) the management of irAE is unclear as potential drugs suppressing these events may also suppress the effectiveness of the immunotherapy itself.

In the current study, we tested autoimmune responses in mouse lung, heart, kidney and colon tissues thirty days following an effective tumor vaccination combined with immune checkpoint inhibitors. Nanostring AutoImmune mouse gene profiling and immunohistochemistry staining were used to analyze signaling pathways, immune cell infiltrates and changes in gene profiles. We found that there were varying levels of immune cell infiltration in the heart, lungs and kidneys. In addition, multiple autoimmune signaling pathways were significantly upregulated, especially in the heart and kidneys. Surprisingly, there was no obvious autoimmune response in the colon of mice tested, which was unexpected considering the higher incidence of colitis seen in patients receiving immune therapy. Speculatively, the colitis may be pre-conditioned in patients and exacerbated by immunotherapy which does not seem to be the case in the mouse model. These results suggest that the tumor vaccine model induced organ specific autoimmune responses, that were clinically occult and preferentially detected in cardiac and renal tissues. The results are based on 770 human genes encompassing 35 pathways and processes, that are involved in immune system dysfunction and autoimmune disease. The cell profiling feature included in the panel allows for the relative quantification of 14 different immune cell types (23). The score of total lymphocytes (CD45+ cells), cytotoxic T cells (CD8α+ cells), exhausted T cells, Treg, neutrophils, DCs and mature NK cells are all significantly increased in the cardiac tissue collected from vaccinated mice when compared to naïve and tumor only controls. The scores of B cells, mast cells and macrophages didn’t change significantly. The ratio of CD8+ T cells/Total TIL, and CD8+ T cells/CD4+ T cells were also enhanced following vaccination. Similar trends of T cells (cytotoxic, exhausted and Treg) and NK cells to a lesser degree were observed in kidney and lung tissue after vaccination. In addition, the score of macrophages and B cells were significantly increased in lung tissue. The signature scores are determined using the Inflammation Signature Algorithm across all RNA input levels. The leukocyte marker CD45 was evaluated using IHC in cardiac tissue from the same animals. Staining confirmed the findings of the nanostring analysis in which the pro-inflammatory infiltration of CD45 following vaccination was detected in the heart tissue which was inhibited by CD24-Fc administration. Although there is moderate lymphocytic infiltration in the major organs examined and immune signatures were upregulated, no obvious life-threatening cardiac or renal side effects were noted in the mice at 30 days, nor were any seen in prior mouse models with long term survival at the carefully selected doses of immune checkpoints.

A CD24-Fc fusion protein composed of the extracellular part of CD24 and the human IgG-Fc portion has shown encouraging results in clinical trials as a specific modulator of auto-inflammatory syndromes. The current study evaluates whether CD24-Fc could be used to prevent or treat irAE while evaluating the effect on T cell anti-tumor immunity with tumor vaccine therapy. We studied two vaccine strategies in mouse neuroblastoma models to evaluate the influence of CD24-Fc on the therapeutic effect of the vaccine. We compared the treatment of CD24-Fc administered at the onset of vaccination (initial) or administered with follow-up booster vaccination (delayed). When CD24-Fc was used at the onset of vaccination, it appeared to have dampened the anti-tumor efficacy of the cancer vaccine. However, when we delayed administration of CD24-Fc for three days at the time of booster vaccination, there was no impact on survival rate in which tumor rejection was similar to controls. The impact on vaccination of initial administration may be explained by the potential immune checkpoint inhibitory and anti-inflammatory function of CD24. Upon administration, CD24-Fc immediately binds to injured tumor cell components and prevents the interaction of DAMPs with toll-like receptors (TLRs) inhibiting both nuclear factor-kappa B (NFkB) activation and secretion of inflammatory cytokines, which may dampen tumor immunity induced by the vaccine (24). In addition, CD24-Fc could bind to and activate Siglec G/10, stimulate SHP-1-mediated inhibitory signaling, and prevent NFkB activation and secretion of inflammatory mediators, which may further prevent lymphocytic infiltration (25). Moreover, CD24-Fc may also initiate tumor cell proliferation and behave as a “don’t eat me” signal to assist in tumor evasion from phagocytosis. On the other hand, using CD24-Fc later with booster vaccination would avoid the initial suppressive effect, thus allowing the vaccine to activate immune pathways and boost immune cell infiltrates in the tumor. Delaying CD24-Fc administration at the time of the booster would theoretically have the desired effect of suppressing DAMPs, limiting non-specific tissue damage and suppressing inflammation through blockade of NF-κB activation (8) without suppressing the anti-tumor effect. Further optimizing the dose and timing of administration of CD24-Fc with vaccination is important for suppressing auto-immunity.

Another interesting finding from this study is the variable effect of CD24-Fc as it was more efficient in suppressing the auto-immune response in the heart when compared to other organs. A caveat to this observation is that the auto-immunity in the heart was most prominent in comparison, thus any suppressive effect would be more obvious. Alternatively, the strong suppressive effect could be due to CD24 receptor engagement. CD24 can interact with Siglecs, a class of sialic acid binding receptors on immune cells and selectively repress tissue damage-induced immune responses. DAMPs such as HMGB1, HSP70 and -90 are presented to Siglec by binding to their high affinity ligand CD24, which leads to the activation of immunoreceptor tyrosine-based inhibitory motifs (ITIM) and the subsequent abrogation of inflammatory cytokine signals through a blockade of NF-κB activation (26). Siglec-G is the major receptor for CD24-Fc in mice (27), and is primarily expressed on immune cells including monocytes, granulocytes and lymphocytes. Our tumor vaccine induced the most abundant immune cell infiltrate in cardiac tissue, which provides CD24-Fc with the most Siglec binding sites and thus a positive feedback loop for CD24-Fc to inhibit the autoimmune response. CD24 Fc can bind to multiple Siglec receptors, and each Siglec has a unique specificity for sialylated ligands, making it more probable that additional signaling pathways, including but not limited to the axis of the CD24-Fc-siglec G may be recruited. It will be of interest to define which ligands and Siglecs are present and able to interact with CD24-Fc in the various organs systems.

Taken together, our data demonstrates that the combination of Myc-overexpressing tumor cell vaccine with check point inhibitors is an efficient and relatively safe therapeutic strategy for treating neuroblastoma in a mouse model. Despite this seemingly safe therapy, occult auto-immune effects are detected in the cardiac, renal and pulmonary tissue evaluated thirty days after vaccination. The systemic administration of CD24-Fc, is sufficient to suppress autoimmune responses in the heart, but appropriate timing of administration is critical in order to avoid suppression of the vaccine therapy effect as noted in this mouse neuroblastoma tumor model.

## Data availability statement

The datasets presented in this study can be found in online repositories. The names of the repository/repositories and accession number(s) can be found in the article/**Supplementary Material**.

## Ethics statement

The animal study was reviewed and approved by the IACUC at Children’s National Hospital.

## Author contributions

AS conceived the idea and acquired funding for the study. AS and XW wrote the original draft, and all authors reviewed and edited the manuscript; XW, MB, PS, TZ, SL, PZ, YL and AS interpreted the data, made the figures, planned the experiments, performed and analyzed the experiments. All authors reviewed, edited, and approved the final manuscript.

## Supplementary material

Supplementary Figure 1 The lung, heart, kidney and colon were harvested from naïve mice (n=3), unvaccinated tumor only mice (n=3), and vaccinated mice (n=12) after tumor inoculation at day 30. The global expression of mRNA from each organ was investigated using NanoString Autoimmune Profiling arrays. Profiling analysis revealed that heart, kidney and lung from the vaccination group demonstrated a moderate to severe increase in signature markers for dendritic cells, neutrophils, macrophages, and B cells when compared to naïve and tumor only control mice. Statistical significance was determined by unpaired two-tailed Student’s t-test, and p<0.05 was considered statistically significant.

Supplementary Figure 2 The autoimmune signaling pathway scores that related to major inflammatory signaling pathways were all significantly augmented in the heart, kidney and lung tissue collected from tumor vaccinated mice, but these were not observed in the colon. Unpaired two-tailed Student’s t-test was performed for the statistical analysis and p<0.05 was considered statistically significant.

Supplementary Figure 3 Delayed CD24Fc treatment did not have significant impact on infiltration of dendritic cells, neutrophils, macrophages, and B cells in heart, kidney, lung and colon tissue. Statistical significance was determined by unpaired two-tailed Student’s t-test and p<0.05 was considered statistically significant.

Supplementary Figure 4 Delayed CD24Fc treatment repressed the pathway scores of multiple autoimmune signals related to lymphocyte differentiation and trafficking in heart tissue. Statistical significance was determined by unpaired two-tailed Student’s t-test and p<0.05 was considered statistically significant.

Supplementary Figure 5 Delayed CD24Fc treatment repressed the pathway scores of multiple autoimmune signals related to chemokines and cytokines in heart tissue. Statistical significance was determined by unpaired two-tailed Student’s t-test and p<0.05 was considered statistically significant.

Supplementary Figure 6 Delayed CD24Fc treatment repressed the pathway scores of multiple autoimmune signals that related to major inflammatory pathways in heart tissue. Statistical significance was determined by unpaired two-tailed Student’s t-test and p<0.05 was considered statistically significant.
