# Supplementary figures and images for: CD24-Fc suppression of immune related adverse events in a therapeutic cancer vaccine model of murine neuroblastoma
Source: Front Immunol. 2023 Jun 6;14:1176370. doi: 10.3389/fimmu.2023.1176370 (PMC10279976; doi:10.3389/fimmu.2023.1176370)

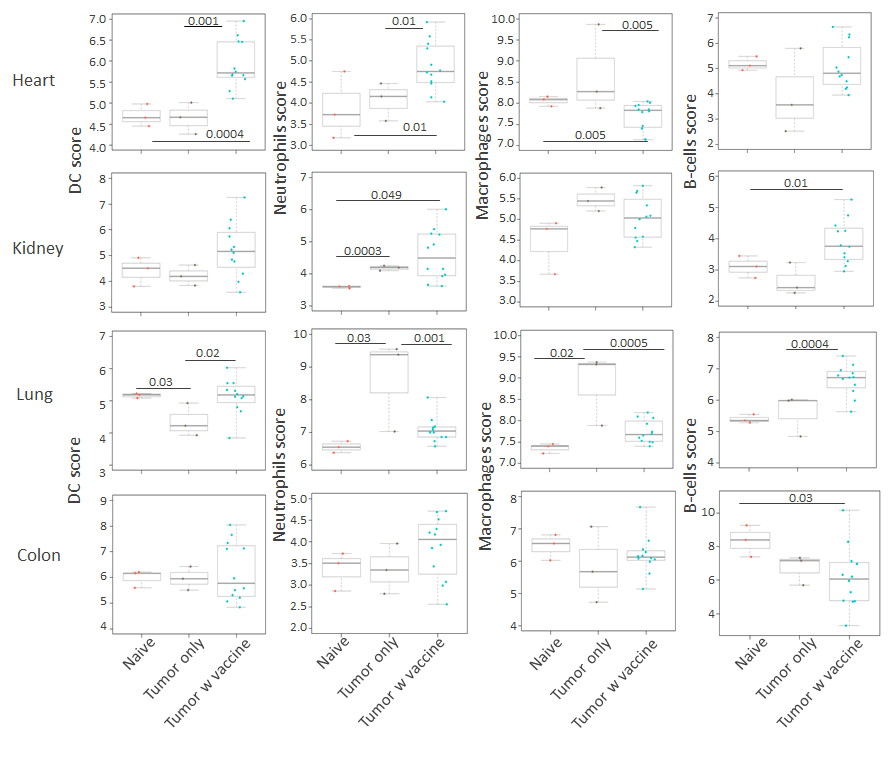

Supplement: Supplementary Figure 1 — The lung, heart, kidney and colon were harvested from naïve mice (n=3), unvaccinated tumor only mice (n=3), and vaccinated mice (n=12) after tumor inoculation at day 30. The global expression of mRNA from each organ was investigated using NanoString Autoimmune Profiling arrays. Profiling analysis revealed that heart, kidney and lung from the vaccination group demonstrated a moderate to severe increase in signature markers for dendritic cells, neutrophils, macrophages, and B cells when compared to naïve and tumor only control mice. Statistical significance was determined by unpaired two-tailed Student’s t-test, and p<0.05 was considered statistically significant. [file Image_1.tif]

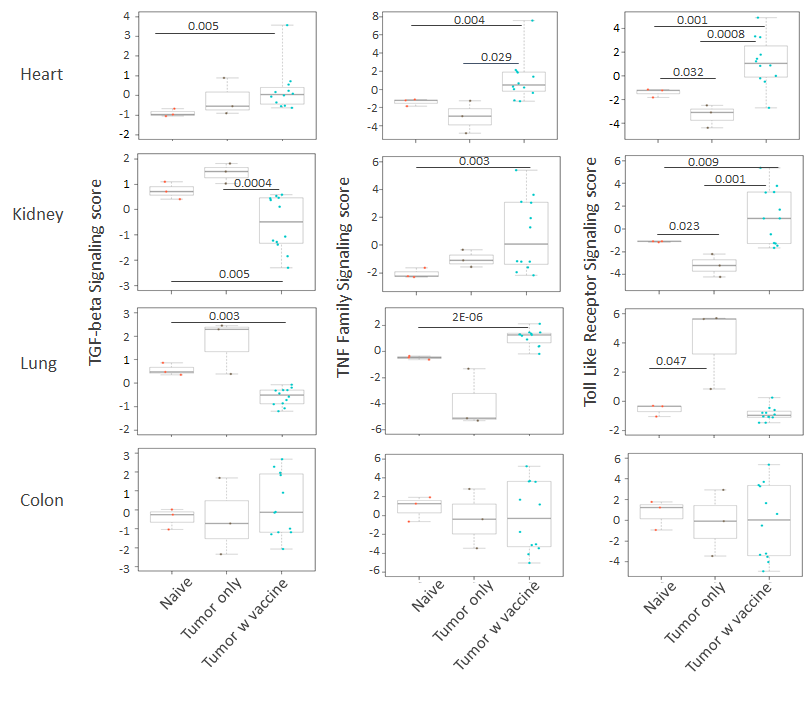

Supplement: Supplementary Figure 2 — The autoimmune signaling pathway scores that related to major inflammatory signaling pathways were all significantly augmented in the heart, kidney and lung tissue collected from tumor vaccinated mice, but these were not observed in the colon. Unpaired two-tailed Student’s t-test was performed for the statistical analysis and p<0.05 was considered statistically significant. [file Image_2.tif]

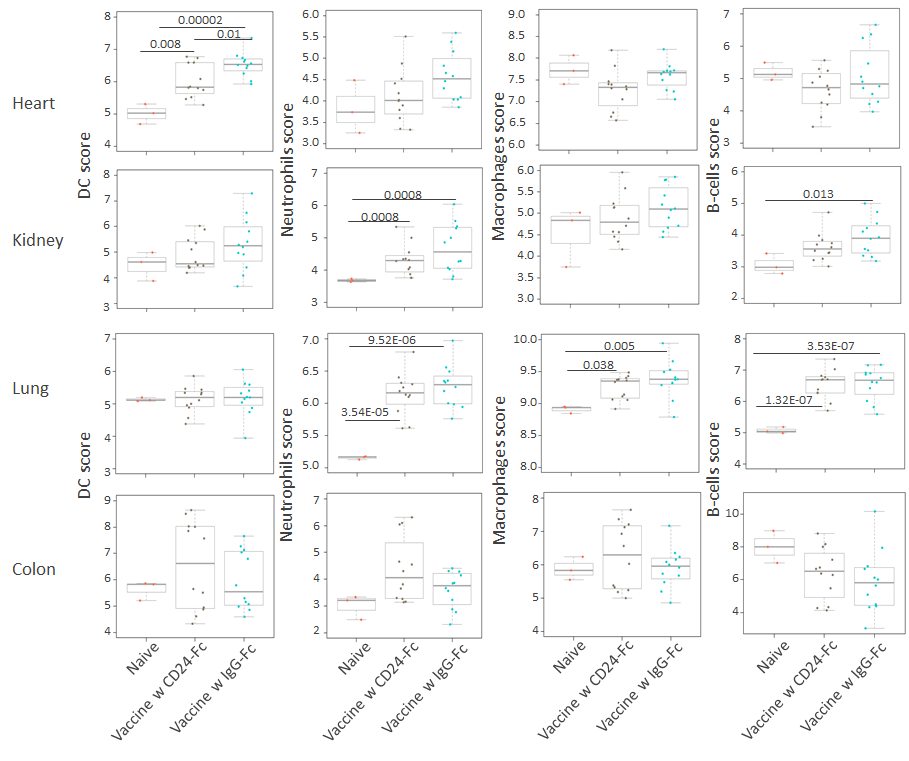

Supplement: Supplementary Figure 3 — Delayed CD24Fc treatment did not have significant impact on infiltration of dendritic cells, neutrophils, macrophages, and B cells in heart, kidney, lung and colon tissue. Statistical significance was determined by unpaired two-tailed Student’s t-test and p<0.05 was considered statistically significant. [file Image_3.tif]

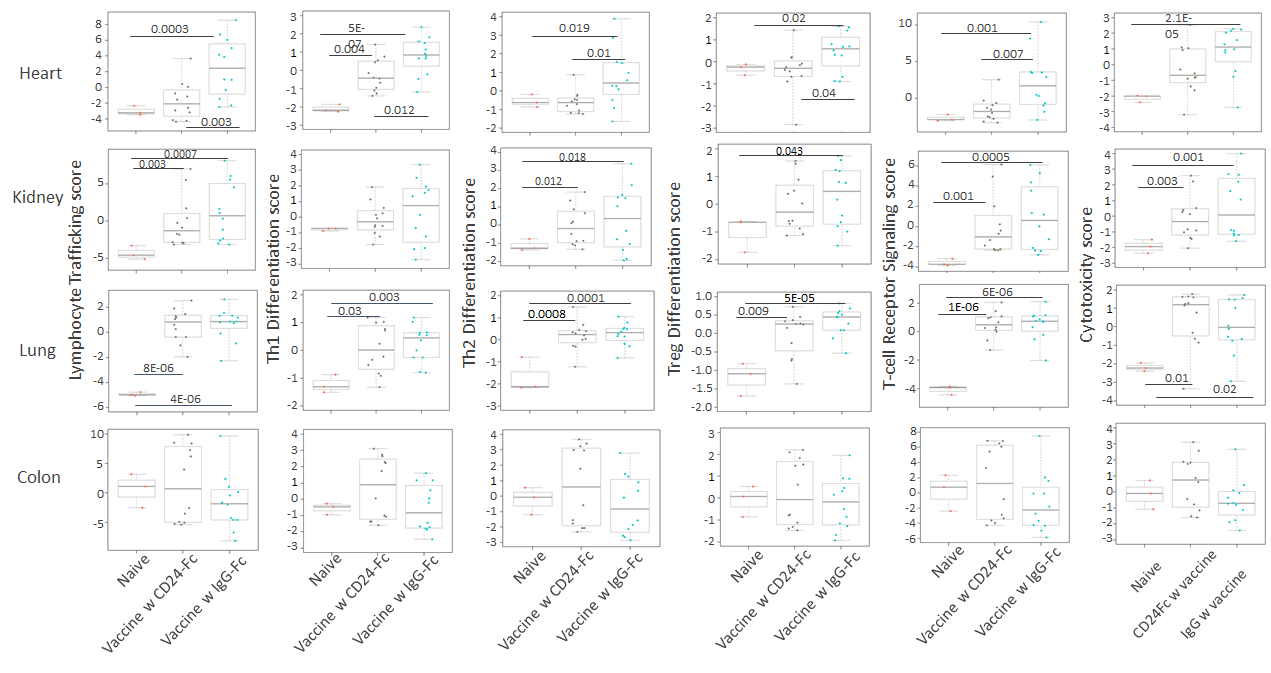

Supplement: Supplementary Figure 4 — Delayed CD24Fc treatment repressed the pathway scores of multiple autoimmune signals related to lymphocyte differentiation and trafficking in heart tissue. Statistical significance was determined by unpaired two-tailed Student’s t-test and p<0.05 was considered statistically significant. [file Image_4.tif]

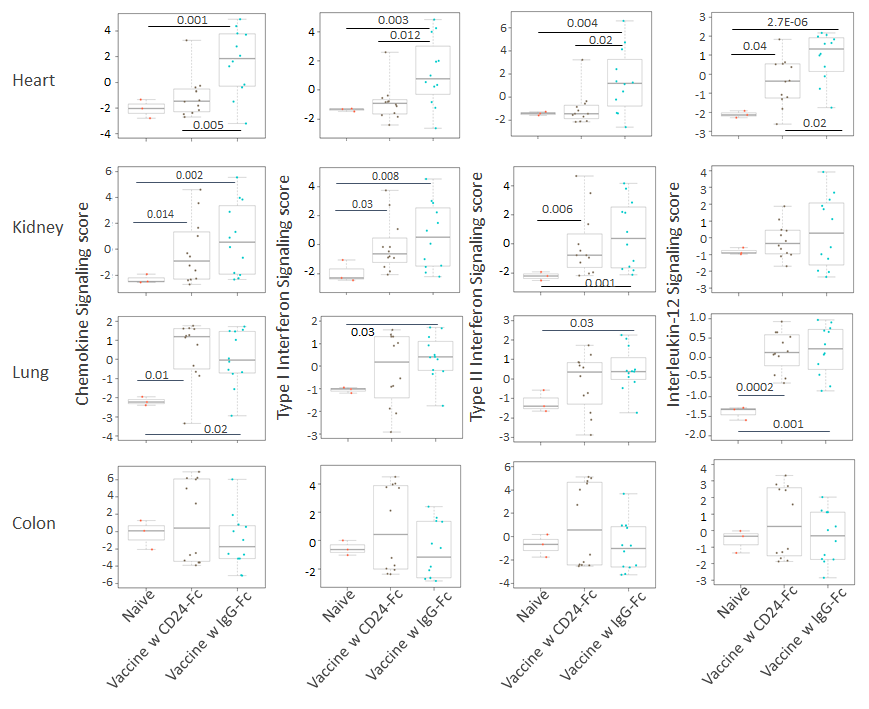

Supplement: Supplementary Figure 5 — Delayed CD24Fc treatment repressed the pathway scores of multiple autoimmune signals related to chemokines and cytokines in heart tissue. Statistical significance was determined by unpaired two-tailed Student’s t-test and p<0.05 was considered statistically significant. [file Image_5.tif]

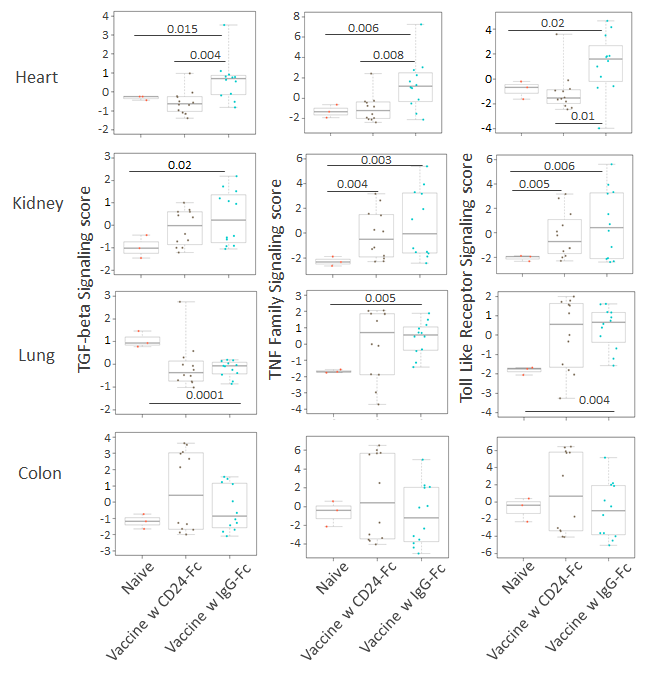

Supplement: Supplementary Figure 6 — Delayed CD24Fc treatment repressed the pathway scores of multiple autoimmune signals that related to major inflammatory pathways in heart tissue. Statistical significance was determined by unpaired two-tailed Student’s t-test and p<0.05 was considered statistically significant. [file Image_6.tif]
